# Influence of socio-contextual factors on the link between traditional and new media use, and young people’s sexual risk behaviour in Sub-Saharan Africa: a secondary data analysis

**DOI:** 10.1186/s12978-024-01868-0

**Published:** 2024-09-30

**Authors:** Helen Uche Okoye, Elizabeth Saewyc

**Affiliations:** School of Nursing, University of British Columbia, T201-2211 Wesbrook Mall, Vancouver, BC V6T 2B5 Canada

## Abstract

**Background:**

Traditional and new media use links to young people’s sexual risk behaviour. The social contexts of young people’s daily lives that influence media use and sexual risk behaviour are often investigated as independent causal mechanisms. We examined the link between media use and young people’s sexual risk behaviour, considering the intersecting socio-contextual factors in Sub-Saharan Africa.

**Methods:**

Age-adjusted bivariate logistic regression models tested the association between traditional media (TV, radio, and newspapers), and new media (mobile phone and online) use and sexual risk behaviour using the Demographic and Health Surveys from six Sub-Saharan African countries among unmarried sexually active youths, aged 15–24 years. Multivariate logistic regression models ascertained the media sources that had an additional influence on young people’s sexual risk behaviour, after accounting for socio-contextual factors, and knowledge about HIV and other sexually transmitted infections.

**Results:**

Socio-contextual factors attenuated the association between media use and young people’s sexual risk behaviour in many countries. However, those who did not have access to new and traditional media were more likely to use unreliable contraceptive methods or not use contraception. Adolescents in Nigeria who did not own phones were 89% more likely to use unreliable contraceptive methods or not use any methods [(AOR = 1.89 (1.40–2.56), p < .001)], those in Angola who did not read newspapers had higher odds of not using contraception or used unreliable methods [(aOR = 1.65 (1.26–2.15), p < .001)]. Young people in Angola (aOR = 0.68 (0.56–0.83), p < .001), Cameroon [(aOR = 0.66 (0.51–0.84), p < .001)], Nigeria [(aOR = 0.72 (0.56–0.93), p = .01)], and South Africa [(aOR = 0.69 (0.49–0.98), p = .03)] who did not own phones were less likely to have 2 or more sexual partners compared to those who owned phones. Lack of internet access in Mali was associated with lower odds of having 2 or more sexual partners (aOR = 0.45 (0.29–0.70), p < .001). Traditional media use was significantly associated with transactional sex in many countries.

**Conclusions:**

Media use is linked to sexual risk behaviour among young people in Sub-Saharan Africa. Socioeconomic inequalities, levels of globalization, as well as rural–urban disparities in access to media, underscore the need to deliver tailored and targeted sexual risk reduction interventions to young people using both traditional and new media.

## Plain language summary

In addition to television, radio, magazines, and newspapers, young people in Sub-Saharan Africa are increasingly using their phones and the Internet to connect with their peers. However, these media sources can lead to sexual risk behaviour because of access to inappropriate and inaccurate information. Social environmental factors in Sub-Saharan Africa, such as gender, place of residence, and socio-economic status influence young people’s access to and use of these media sources. However, many studies that consider the influence of these social factors on the use of media among young people evaluate each of them and fail to consider that these social forces jointly influence young people’s access to the internet, and ability to own phones, watch television, or read newspapers. Our study considered these combined social factors and their influence on the link between the use of media and sexual risk behaviour among young people. We found that considering these social environmental factors reduced the association between media use and young people’s sexual risk behaviour but those who did not have access to these media sources were more likely to use unreliable methods to prevent pregnancy or sexually transmitted diseases. Those who listened to the radio, or read newspapers and magazines were more likely to give or receive cash or other gifts in exchange for sex. In contrast, young people who never owned phones or lacked access to the internet were less likely to have had multiple sexual partners. Our findings stress the need to consider social contexts and media use in promoting young people’s sexual health.

## Introduction

Sexual risk behaviour among young people remains a major public health concern in Sub-Saharan Africa, where risky sexual behaviour and related adverse health outcomes are disproportionately higher compared to high-income countries [1–3]. The transitional changes in the biological, cognitive, psychosocial, and emotional domains during adolescence predispose young people to sexual and reproductive risk behaviour [4–6]. Further, longer school years, and later age of marriage, as well as social transformations in many countries in Sub-Saharan Africa elevate sexual risk behaviour among unmarried young people [7, 8]. Additionally, globalization, and the information, communication, and technology (ICT) revolution of the twenty-first century facilitate easy access to digital sexual and reproductive health information and misinformation among young people in Sub-Saharan Africa [9, 10]. Young people are growing up in a highly digitalized world, where new media sources, such as mobile applications, social media networks, websites, and other internet-related forms of communication have transformed information access and social connections among youth [11–13].

New media refers to internet-based applications that permit access to videos, digital texts, images, associated web links, and user interactions [9, 14, 15]. Available evidence in Sub-Saharan Africa indicates that most of the internet users are young people, thus, new media use constitutes emerging social contextual factors that can have significant impacts on sexual and reproductive health during this critical stage in the life course [16–18]. Indeed, uncontrolled access to inappropriate pornographic materials on digital media among young people in Sub-Saharan Africa is linked to sexual and reproductive risk behaviour [9, 19–21]. For example, sexting, or sharing sexually explicit materials through mobile phones is common among youths and is linked to a higher number of sexual partners and other sexual risk behaviour [17, 22, 23]. Mobile phones and the internet are common new media utilized by young people in Sub-Saharan Africa to access digital information and thus present opportunities for the delivery of mobile sexual and reproductive health interventions [24, 25].

Consequently, many programs and interventions that focus on promoting youths’ sexual and reproductive health in Sub-Saharan Africa leverage the ubiquitous use of new media, particularly mobile phones by young people to communicate factual and comprehensive sexual health information [14, 18, 26, 27]. Indeed, the digital age has revolutionized the dissemination of sexual health messages, and HIV risk awareness campaigns, and increased the uptake of sexual risk preventive services in the region [28–30]. Moreover, young people take advantage of the proliferation of new media and the availability of sexual information on the internet, and social networking sites (e.g., Facebook, YouTube, WhatsApp, Myspace, and Twitter) at no cost to search for answers to sensitive sexual health topics [13, 26, 31]. Thus, young people use new media as alternative sources of sexual health information to circumvent unfriendly sexual health services in the region and to meet their sexual health information needs [26, 32–36].

Besides, the new media, traditional media (such as television, radio, newspapers, and magazines) are sources of sexual and reproductive health, and HIV-related information in Sub-Saharan Africa [37–40]. For example, a study carried out in Nnewi, Nigeria, found that 40% of young people sought information about sexual issues online, another 30% obtained similar information from television, and 7% from newspapers and magazines [41]. Another study in Kenya that investigated the influence of television on adolescents’ sexual behaviour reports that young people were attracted to television programs with sexual content and would rather seek sexual information from the television rather than from their parents or other adults [42].

Given the curiosity and exploration that occur during these developmental years, studies suggest that young people in Sub-Saharan Africa access uncensored sexually explicit materials that are easily available on the internet [15, 17, 21]. A body of evidence reports a positive correlation between media use and a higher likelihood of sexual risk behaviour (such as multiple sexual partners, transactional sex, and non-use or unreliable contraception) among young people in the region [20, 31, 38, 43–45]. For example, a study carried out in Osogbo, Nigeria found that, out of the 68% of young people who owned smartphones, only about 39% used them to access sexual health information and 78% of young people used their phones to access sexually explicit materials [16]. Another study that examined the predictors of risky sexual behaviour among pre-college students in Ethiopia found that social media users had higher odds of having engaged in sexual risk behaviour compared to non-users [45]. Such risky behaviours lead to unsafe abortions, early pregnancy, pregnancy-related complications, intergenerational poverty among girls, and predispose young people to HIV/AIDS [46–48].

However, not all young people in Sub-Saharan Africa have access to new and traditional media sources because of the social circumstances of their daily lives [10, 49–51]. Socio-contextual factors (such as gender, educational attainment, religion, socio-economic status, and place of residence) play a key role in access to and use of media as well as engagement in sexual risk behaviour [43, 49, 52, 53]. From an intersectional theoretical standpoint, the broader social forces (e.g., policies, laws, national wealth, and its distribution), as well as power imbalances place individuals and groups at structural disadvantages and social deprivations [54–57]. Consequently, many young people may not be reached by intervention efforts that aim to address their sexual health information needs through digital health platforms and traditional media sources [10, 24, 49]. Further, poverty and lack of access to formal education expose many young people to sexual risk behaviour [10, 58, 59]. For instance, adolescent girls and young women’s desire to own a mobile phone to earn social status, gain peer approval, or overcome economic vulnerabilities is also related to engagement in transactional sex [60–63].

Additionally, there is conflicting evidence about the role that social factors play in the link between media use and risky sexual behaviour among young people in Sub-Saharan Africa. For example, one study that pooled the Demographic and Health Survey datasets from 27 Sub-Saharan African countries reported that adolescent girls and young unmarried women, who listened to the radio, lived in urban areas, belonged to high social class, or were employed had higher odds of having had a sexually transmitted infection [38]. The same study found that ever attending school, or exposure to television correlated with lower odds of reporting an STI [38]. Another study in Mali reported that mass media exposure had a positive correlation with self-reported sexually transmitted infections [64]. Moreover, many studies in Sub-Saharan Africa examine these social forces as independent causal mechanisms and fail to account for the interacting social factors that influence the link between media use and adolescent sexual risk behaviour [65, 66]. To fill these important gaps in the literature, we explored the link between media use and adolescent sexual risk behaviour in Sub-Saharan Africa while considering the simultaneous influence of other socio-contextual factors.

Specifically, we addressed the following research questions; (1) What is the association between media use and young people’s sexual risk behaviour, over and beyond other social factors? (2) How do young people’s HIV and other STIs knowledge modify the influence of media on their sexual risk behaviour? (3) What are the between-country and regional differences in the link between media use and young people’s sexual risk behaviour?

## Methods

### Design of the study and data source

Our study was a secondary analysis of the publicly available Demographic and Health Surveys (DHS) datasets (2014–2018) from six Sub-Saharan African countries. Demographic and Health Surveys use nationally representative samples to monitor population health. The surveys are carried out every five years by the DHS Program, an agency of the United States Agency for International Development (USAID) in collaboration with participating countries. The DHS uses a cross-sectional design and two-stage sampling to select clusters and households, and an interviewer-administered questionnaire collects information from participants, aged 15–49 in selected households. The DHS has standard model questionnaires that collect health information separately from men, women, and entire households [67]. For the current study, we merged the men's and women’s data files.

More details about the DHS and its administration are provided elsewhere [43, 68]. Most of the questionnaire items that address the socio-demographic characteristics, HIV, and other sexual risks related knowledge, as well as sexual risk behaviour among young people in SSA, are comparably the same for most Sub-Saharan African countries and thus allow for between-country comparisons. We followed the Strengthening the Reporting of Observational Studies in Epidemiology (STROBE) checklist in our study and in reporting our findings.

### Sampling and inclusion criteria

We selected the sub-sample of all the unmarried sexually active young people, aged 15–24 years, in countries that met our inclusion criteria. We considered the national income classification, geographical location of the country in Sub-Saharan Africa, data availability, and consistency of the items that assessed traditional and new media use among young people. We included countries that had measures for phone ownership, access to the internet, and traditional media sources, as well as measures of sexual risk behaviour among young people. For statistical power and to allow for between-country comparisons of adolescent sexual risk behaviour, we selected countries with a population of twenty million or more. We used the World Bank’s Classification of Sub-Saharan African countries based on their Gross National Income (GNI) to categorize these countries into low-income, lower-middle-income, and upper-middle-income countries. This will facilitate an understanding of how the link between media use and sexual and reproductive risk behaviour is similar or different across countries in the region. The countries we included in the study were Angola, Ethiopia, Cameroon, Mali, Nigeria, and South Africa.

### Study variables

#### Outcome variables

Engagement with multiple sexual partners, transactional sex, and contraceptive use (non-use, the use of unreliable contraceptive methods, and the use of modern methods of contraception) among young people were the outcome variables in our study. Our study sample included all unmarried sexually active young people, given their heightened risk of sexual risk behaviour. We used the variable that assessed the number of sexual partners in the 12 months before the survey to assess engagement in sexual risk behaviours. We reasoned that the variable that asked for the number of sexual partners in the last 12 months facilitates easy recall of sexual activity among sexually active young people, so we used the variable that assessed the number of sexual partners in the last 12 months. Since a higher number of sexual partners increases the risk of HIV and other STIs, the number of sexual partners in the 12 months before the surveys was re-coded from a continuous variable to an ordinal level variable (no sexual partner, one sexual partner, two or more sexual partners). Transactional sex was assessed separately for males using the variable that asked whether male youths have ever exchanged cash or other gifts for sex, with females—we used the variable that asked whether they ever had sex in return for gifts, cash, or other material gratification.

The measure of contraceptive method used by the participants was an ordinal level variable (no method, the use of traditional methods, and the use of modern methods of contraception). We dichotomized contraceptive method use at the time of the survey, so that, non-use of any contraceptive method and the use of traditional methods constitute non-use or the use of unreliable methods. While the use of modern methods of contraception represented the use of reliable contraceptive methods. The DHS conceptualizes the traditional methods of contraception as the use of periodic abstinence, coitus interruptus (withdrawal), and other country-specific folkloric methods, such as the use of herbs, amulets, etc. We reasoned that the non-use of contraception, or the use of unreliable methods, for example, folkloric and other methods that have higher failure rates compared to modern methods of contraception could expose young people to unintended pregnancy, as well as sexually transmitted infections including HIV. Further, early pregnancy and unintended pregnancy may result in unsafe abortion, early childbearing, or early marriage [48, 69, 70]. These adverse reproductive outcomes increase morbidity and mortality rates and have lifelong and intergenerational consequences among young people, particularly among unmarried sexually active girls and young women [48, 71]. Moreover, many traditional methods of contraception that are used in Sub-Saharan Africa are not scientifically proven, even the more commonly used methods, e.g., withdrawal or periodic abstinence have higher failure rates and are less effective compared to most modern methods of contraception [72–75]. For instance, one study found no significant difference in preventing unwanted pregnancy among women who used traditional methods compared to those who did not use any contraceptive method [73]. Another study notes that unwanted outcomes, such as unintended births and abortions were higher for non-users of modern methods compared to those who used modern methods [74]. Thus, we used the re-coded binary variable (non-use, or the use of unreliable methods of contraception, and the use of modern methods of contraception) as one of the sexual risk behaviour among young people.

#### Explanatory variables

We included access to the internet and ownership of mobile phones as measures of new media use. These media sources were dichotomous variables that required a yes or no response. The measures of traditional media sources were the frequency of watching television, listening to the radio, and reading magazines and newspapers. The items that measured traditional media use were ordinal level variables (not at all, at least once a week, and almost every day). These measures were dichotomized so that no access to traditional media represented non-use, and the use of these media sources at least once a week and almost every day constituted access to these traditional media sources. We then included several socio-contextual factors that are associated with media use, and risky sexual behaviour among young people in Sub-Saharan Africa [22, 38, 43, 64, 76–78]. We used the DHS classification of the place of residence as urban and rural and the categorization of gender into male and female.

Further, we considered that level of education may influence engagement in sexual and reproductive risk behaviour. For example, available evidence indicates that lower levels of education correlate positively with engagement in sexual risk behaviour among young people [79]. We used an intersectionality lens to categorize young people based on their educational attainment into no education/primary education, and higher/secondary education. Given the prevalent poverty and the precarious social circumstances in Sub-Saharan Africa, young people who never attended school, who had just primary education, or dropped out of school have a higher likelihood of engaging in sexual risk behaviour compared to their counterparts who are enrolled in secondary school or higher [22, 80–83]. For instance, evidence indicates that the odds of condomless sex were lower among young people who had six or more years of schooling [84]. Further, most sex education programs in Sub-Saharan Africa are school-based, and many sexual risk behaviour reduction interventions target young people in high or tertiary education [85]. For example, one systematic review reported that 84% of school-based sexual health education programs to prevent sexually transmitted infections and HIV/AIDS in Sub-Saharan Africa were carried out in secondary and tertiary schools [86].

Socioeconomic status was a proxy variable created from the DHS wealth quintile that incorporates not only participants' income but also inequalities in household characteristics and assets. The wealth quintiles were re-coded into poor, middle class, and rich. Ethnicity was re-coded into least, moderate, and most dominant groups in countries when the ethnic identities could be categorized into distinct groups. We included religious affiliation in all countries except South Africa because the variable was not available in the datasets. Head of the household was a dichotomous variable that categorized households into male- and female-headed households.

#### Ethical consideration

Our study was a secondary analysis of existing anonymous datasets and did not require ethical review.

#### Data analysis

Complex Sample frequencies and percentages described the characteristics of the sample. Rao-Scott’s adjusted chi-square tested the significant association between technology and media use and sexual risk behaviour. Binomial and ordinal logistic regression models tested the relationship between media use and sexual risk behaviour in each country. Next, we sequentially included the social factors in logistic regression models without replacement to assess the change in the influence of each socio-contextual factor on the relationship between media use and young people’s sexual risk behaviour. The multivariate models examined the media sources’ relationship, controlling for all the socio-contextual factors, on each sexual risk behaviour. Last, we included young people’s knowledge about HIV risk and other STIs in the final multivariate model to evaluate the independent influence of new and traditional media use on young people’s sexual risk behaviour, controlling for personal knowledge and socio-contextual factors. Additionally, we ascertained the media sources that had significant independent links to sexual risk behaviours, over and beyond other socio-contextual factors, as well as knowledge about HIV and other STIs. We adjusted for age in the logistic regression models because sexual risk behaviour and media use vary by age [87–92]. In addition, we were interested in understanding the influence of social factors on the link between unmarried young people's use of technology and media, and engagement in sexual risk behaviour. We performed all the data analysis in the Complex Samples Module in SPSS, version 28.0 (IBM Corporation, Armonk, NY, USA), and the level of significance was 0.05.

## Results

### Sociodemographic profile of the study participants

The results in Table 1, reveal that more than half of the young people in Angola, close to two-thirds in Cameroon, and more than 86% in South Africa were unmarried and sexually active. In contrast, only a fifth in Ethiopia and about a fourth in Mali were unmarried and sexually active. A greater percentage of youths in Angola and Mali were between 15 and 19 years old, rather than 20–24 years. More than half of unmarried sexually active young people in Mali and about two-thirds in Cameroon were girls, while more than 77% in Nigeria and 65% in South Africa were girls. A greater majority of young people in Angola, Cameroon, Nigeria, and South Africa had secondary education or higher. A good number of the youths in South Africa had secondary education or higher, and a greater percentage of youths in Ethiopia, Mali, and Nigeria were employed. A greater percentage of young people had access to the internet in Cameroon and South Africa. Across all the countries, most young people had mobile phones, and more than half of them watched television or listened to the radio in all the countries. In contrast, most youths did not read newspapers in all countries, except in South Africa where more than 66% read newspapers.

**Table 1:** Sociodemographic characteristics

	Angola (weighted n = 4145)	Ethiopia (weighted n = 794)	Cameroon (weighted n = 2800)	Mali (weighted n = 944)	Nigeria (weighted n = 3015)	South Africa (weighted n = 2395)
Unmarried sexually active	59.5	20.0	56.0	26.4	30.8	86.4
Age in years (15–19 years)	57.2	34.2	44.0	52.7	41.2	37.6
Girls	41.4	29.0	59.0	52.3	77.4	65.5
Place of residence (Urban)	78.2	39.3	67.3	42.1	55.6	61.4
Secondary education/Higher	67.2	46.6	85.4	55.5	89.6	94.7
Working	43.1	69.5	49.9	62.5	59.7	16.0
Internet access	43.8	32.8	58.5	46.6	47.2	65.8
Mobile phone ownership	63.0	71.7	68.8	81.4	75.7	88.4
Watched television						
Not at all	23.7	36.5	22.3	16.9	24.0	17.1
At least once a week	19.7	26.8	16.1	22.3	29.6	11.1
Almost everyday	56.6	36.7	61.5	60.8	46.4	71.8
Read Newspapers						
Not at all	58.5	63.2	72.3	85.8	71.9	33.3
At least once a week	33.2	23.2	18.8	9.0	18.2	29.4
Almost everyday	8.3	13.6	8.9	5.2	9.9	37.4
Listened to radio						
Not at all	35.6	36.7	49.1	26.9	34.7	28.9
At least once a week	35.7	24.1	28.1	24.5	33.9	19.9
Almost everyday	28.7	39.2	22.8	48.6	31.4	51.2

The frequency distribution of sexual risk behaviours among the youths **(**Table 2**)** indicates a greater percentage of young people had one sexual partner in the 12 months before the survey. About one-fifth of youths in Cameroon had two or more sexual partners, and up to 15% in South Africa had two or more sexual partners. About 70% of the young people in Angola, Mali, and Nigeria did not use any contraception or used unreliable contraceptive methods. Similarly, more than half in Cameroon and 49% in Ethiopia did not use any contraception or used unreliable contraceptive methods.

**Table 2:** Sexual risk behaviour among young people in Sub-Saharan Africa

	Number of sexual partners (Last 12 months)		Contraceptive use	Transactional sex	
	One sexual partner	2 or more sexual partners	Non-use or unreliable contraceptive use	Young women	Young men
		%			
Angola (weighted n = 4145)	71.6	9.4	71.4	1.9	3.2
Cameroon (weighted n = 2800)	67.4	20.2	52.5	10.1	10.6
Ethiopia (weighted n = 794)	55.6	7.9	49.4	0.3	7.7
Mali (weighted n = 944)	70.6	8.6	71.1	13.7	14.2
Nigeria (weighted n = 3015)	70.5	9.6	72.7	19.3	18.1
South Africa (weighted n = 2395)	75.7	15.1	34.1	1.6	2.5

### Association between the use of technology and media and sexual risk behaviour

The age-adjusted logistic regression models on the relationship between technology and media and sexual risk behaviour **(**Table 3**)** show that, in all six countries (Angola, Cameroon, Ethiopia, Mali, Nigeria, and South Africa), lack of access to technology and media was associated with lower odds of having two or more sexual partners. In Angola, youths who did not have access to the internet [(aOR = 0.68 (0.56–0.83), p < 0.001)], those who did not read newspapers [(0.74 (0.59–0.94), p = 0.01)], as well as those who did not own mobile phones [(aOR = 0.59 (0.48–0.72), p < 0.001)] were significantly less likely to have two or more sexual partners. Similarly, in Cameroon, Ethiopia, Mali, Nigeria, and South Africa lack of access to the internet or no mobile phone ownership was significantly associated with lower odds of having two or more sexual partners.

**Table 3:** Age-adjusted bivariate logistic regression media use and sexual risk behaviour

	Technology and media use				
	No Internet use (vs Internet access	No mobile phone (vs. Mobile phone ownership	No TV access (vs TV access)	No radio Access (vs. Radio	Did not read the newspaper (vs Read)
		aOR 95% CI			
**Angola**					
2 or more sexual partners	0.68*** (0.56–0.83)	0.59*** (0.48–0.72)	0.85 (0.71–1.03)	0.89 (0.73–1.09)	0.74* (0.59–0.94)
None or use of unreliable contraceptive methods	3.72*** (3.00–4.61)	3.00*** (2.41–3.73)	5.16*** (3.74–7.13)	2.46*** (1.96–3.08)	3.34*** (2.69–4.14)
Transactional sex					
Young men	2.55** (1.43–4.54)	0.95 (0.55–1.66)	2.65*** (1.62–4.34)	2.66*** (1.67–4.22)	1.93* (1.12–3.32)
Young women	0.97 (0.47–2.01)	1.16 (0.56–2.40)	1.86 (0.97–3.59)	2.77* (1.16–6.58)	1.73 (0.72–4.14)
**Cameroon**					
2 or more sexual partners	0.66** (0.54–0.80)	0.84 (0.66–1.04)	0.86 (0.65–1.14)	0.82 (0.66–1.02)	0.99 (0.79–1.24)
None or use of unreliable contraceptive methods	1.96** (1.64–2.34)	1.02 (0.82–1.27)	1.81*** (1.36–2.41)	1.48*** (1.24–1.76)	0.99 (0.81–1.23)
Transactional sex					
Young men	1.39 (0.94–2.05)	0.69 (0.42–1.13)	1.29 (0.79–2.12)	1.58* (1.04–2.41)	1.15 (0.72–1.86)
Young women	2.25** (1.46–3.48)	2.17** (1.45–3.27)	1.64* (1.10–2.46)	1.30 (0.89–1.90)	1.91** (1.25–2.90)
**Ethiopia**					
2 or more sexual partners	0.67* (0.46–0.97)	0.56* (0.36–0.89)	0.79 (0.50–1.25)	0.99 (0.63–1.56)	0.81 (0.56–1.18)
None or use of unreliable contraceptive methods	2.14** (1.30–3.53)	1.60* (1.02–2.51)	1.67* (1.09–2.55)	1.29 (0.83–2.00)	1.36 (0.90–2.05)
Transactional sex					
Young men	0.82 (0.34–1.99)	0.84 (0.31–2.26)	0.55 (0.20–1.52)	1.89 (0.76–4.71)	1.58 (0.64–3.92)
Young women	0.56 ((0.05–7.05)	0.87 (0.10–7.70)	∞	∞	0.89 (0.16–4.97)
**Mali**					
2 or more sexual partners	0.43*** (0.31–0.59)	0.55** (0.37–0.81)	0.80 (0.53–1.19)	0.93 (0.65–1.32)	0.55** (0.37–0.84)
Non-use/ineffective contraception	2.93*** (2.05–4.19)	2.19** (1.38–3.49)	1.68* (1.02–2.76)	1.54* (1.08–2.19)	2.19*** (1.45–3.31)
Transactional sex					
Young men	0.88 (0.48–1.61)	0.54 (0.19–1.85)	1.39 (0.66–2.91)	0.59 (0.26–1.33)	0.48* (0.24–0.98)
Young women	0.78 (0.38–1.59)	0.50 (0.22–1.10)	0.43 (0.15–1.30)	0.57 (0.24–1.34)	0.44* (0.23–0.84)
**Nigeria**					
2 or more number of sexual partners	0.70** (0.57–0.85)	0.64** (0.53–0.78)	1.42*** (1.67–1.72)	1.70*** (1.40–2.08)	0.94 (0.77–1.16)
Non-use/ineffective contraception	2.15** (1.76–2.62)	2.87** (2.24–3.68)	1.60*** (1.27–2.02)	1.42*** (1.17–1.72)	1.70*** (1.40–2.08)
Transactional sex					
Young men	1.08 (0.71–1.63)	1.30 (0.77–2.20)	1.71* (1.07–2.73)	1.54 (0.92–2.59)	1.74* (1.05–2.88)
Young women	1.28 (0.87–1.87)	1.33 (0.98–1.81)	0.99 (0.71–1.40)	1.52** (1.15–2.01)	1.97*** (1.34–2.91)
**South Africa**					
2 or more sexual partners	0.81 (0.64–1.02)	0.69* (0.48–0.97)	0.99 (0.75–1.31)	0.99 (0.77–1.27)	0.87 (0.69–1.10)
Non-use/ineffective contraception	1.06 (0.87–1.30)	1.14 (0.83–1.58)	1.10 (0.86–1.43)	1.20 (0.97–1.49)	1.09 (0.89–1.35)
Transactional sex					
Young men	1.86 (0.73–4.74)	0.85 (0.26–2.30)	2.36 (0.68–8.20)	3.94** (1.53–10.26	0.24* (0.07–0.84)
Young women	2.03* (1.02–4.06)	2.24 (0.71–7.07)	2.15 (0.66–7.04)	2.66* (1.09–6.47	2.28 (0.87–5.97)

*aOR* age-adjusted odds ratio Blank spaces denote non-significant chi-square test of difference

*P < .05 **P < .01 ***P < .001

On the other hand, in five out of the six countries, lack of access to the internet and no ownership of mobile phones was associated with higher odds of non-use or the use of unreliable methods of contraception. Lack of access to traditional media sources was also associated with higher odds of unreliable contraception or non-use of any contraceptive method in these five countries. In Cameroon, young people who did not have access to the internet, did not watch television, and did not listen to the radio were 96% (aOR = 1.96 (1.64–2.34), p = 0.000), 81% (aOR = 1.81 (1.36–2.41), p < 0.001), and 48% (aOR = 1.48 (1.24–1.76), p < 0.001) more likely to use unreliable contraceptive methods or not use any contraception at all. While lack of access to or non-use of both traditional and new media was significantly associated with engagement in transactional sex among both young women and young men in many countries, young men in Mali (aOR = 0.48 (0.24–0.98), p = 0.04) and South Africa (aOR = 0.24 (0.07–0.84), p = 0.02) who did not read newspapers were significantly less likely to report transactional sex. There was no significant association between technology and media use and engagement in transactional sex among young people in Ethiopia.

### Relationship between new and traditional media use and sexual risk behaviour adjusting for socio-contextual factors and HIV and other STIs knowledge

The multivariate models reveal that socio-contextual factors modified the relationship between technology and media use and young people’s sexual risk behaviour. In many countries, the socio-economic class of families, young people’s place of residence, religion, educational attainment, and employment status significantly influenced the link between media use and sexual risk behaviour. However, ownership of phones had an independent influence on having multiple sexual partners in four out of the six countries, after adjusting for socio-contextual factors. This relationship persisted even after adjusting for HIV and other STIs knowledge **(**Table 4**).** Those who did not own a mobile phone were significantly less likely to have two or more sexual partners in Angola (aOR = 0.68 (0.55–0.83), p < 0.001); Cameroon (aOR = 0.66 (0.51–0.84), p < 0.001); Nigeria (aOR = 0.72 (0.56–0.93), p = 0.01), and South Africa (aOR = 0.69 (0.49–0.98), p = 0.03). In Mali, lack of access to the internet was significantly associated with lower odds of having had two or more sexual partners (aOR = 0.45 (0.29–0.70), p < 0.001).

**Table 4:** Relationship between media use and sexual behaviour adjusting for social factors, HIV and other STIs knowledge

	Technology and media use				
	No Internet use (vs Internet access	No mobile phone (vs. Mobile phone ownership	No TV access (vs TV access)	No radio Access (vs. Radio	Did not read newspaper (vs Read)
Sexual risk behaviour	AOR 95% CI				
**Angola**					
2 or more sexual partners	0.79 (0.60–1.04)	0.68*** (0.55–0.83)	1.03 (0.77–1.38)	1.10 (0.87–1.40)	0.82 (0.62–1.09)
None or use of unreliable contraceptive methods	1.35 (0.99–1.85)	1.18 (0.87–1.59)	1.33 (0.88–2.01)	0.87 (0.66–1.15)	1.65*** (1.26–2.15)
Transactional sex					
Young men	1.30 (0.65–2.59)	0.53 (0.23–1.25)	0.90 (0.50–1.61)	1.51 (0.86–2.65)	1.32 (0.60–2.87)
Young women	0.37 (0.09–1.42)	0.71 (0.36–1.41)	0.89 (0.42–1.90)	2.71 (0.94–7.80)	1.32 (0.52–3.37)
Unemployed					
**Cameroon**					
2 or more sexual partners	0.80 (0.64–1.00)	0.66*** (0.51–0.84)	1.04 (0.74–1.46)	0.95 (0.76–1.19)	1.09 (0.85–1.41)
None or use of unreliable contraceptive methods	1.19 (0.96–1.48)	1.24 (0.95–1.63)	1.08 (0.75–1.56)	1.19 (0.97–1.43)	0.77* (0.60–0.99)
Transactional sex					
Young men	0.91 (0.48–1.73)	0.53* (0.31–0.93)	0.61 (0.30–1.26)	1.62 (0.90–2.93)	0.75 (0.39–1.46)
Young women	1.52 (0.94–2.46)	1.49 (0.99–2.25)	0.84 (0.47–1.51)	1.09 (0.73–1.65)	1.36 (0.87–2.13)
**Ethiopia**					
2 or more sexual partners	0.91 (0.56–1.49	0.69 (0.42–1.15)	1.06 (0.61–1.86)	1.17 (0.69–1.98)	0.95 (0.63–1.44)
None or use of unreliable contraceptive methods	1.68 (0.86–3.26)	0.97 (0.56–1.69)	1.19 (0.64–2.21)	0.81 (0.45–1.44)	0.88 (0.52–1.49)
Transactional sex					
Young men	0.64 (0.24–1.73)	1.30 (0.48–3.50)	0.18* (0.05–0.69)	3.60** (1.38–9.40)	1.85 (0.71–4.84)
Young women	∞	∞	∞	∞	∞
**Mali**					
2 or more sexual partners	0.45*** (0.29–0.70)	0.76 (0.49–1.18)	1.16 (0.69–1.94)	1.07 (0.70–1.64)	0.64 (0.39–1.07
None or use of unreliable contraceptive methods	1.43 (0.94–2.21)	1.20 (0.69–2.10)	0.78 (0.45–1.36)	1.12 (0.74–1.71)	1.43 (0.91–2.23)
Transactional sex					
Young men	1.15 (0.52–2.56)	0.77 (0.20–2.91)	2.31 (0.96–5.57)	0.41* (0.18–0.94)	0.56 (0.25–1.26)
Young women	1.87 (0.85–4.10)	0.55 (0.20–1.49)	0.59 (0.14–2.44)	0.71 (0.28–1.84)	0.65 (0.34–1.40)
**Nigeria**					
2 or more sexual partners	0.79 (0.61–1.02)	0.72* (0.56–0.93)	1.10 (0.87–1.38)	1.13 (0.92–1.40)	1.06 (0.85–1.32)
None or use of unreliable contraceptive methods	1.17 (0.92–1.50)	1.89*** (1.40–2.56)	1.11 (0.82–1.51)	1.03 (0.80–1.32)	1.56 (0.91–1.46)
Transactional sex					
Young men	0.67 (0.41–1.100	1.17 (0.63–2.18)	1.15 (0.61–2.18)	1.11 (0.58–2.13)	1.73 (0.96–3.13)
Young women	1.15 (0.75–1.76)	1.30 (0.89–1.89)	0.76 (0.50–1.16)	1.44* (1.04–1.99)	1.77** (1.67–2.69)
**South Africa**					
2 or more sexual partners	0.98 (0.73–1.31)	0.69* (0.49–0.98)	1.22 (0.88–1.68)	1.16 (0.90–1.49)	0.91 (0.72–1.17)
None or use of unreliable contraceptive methods	0.88 (0.70–1.11)	1.27 (0.92–1.74)	0.93 (0.69–1.25)	1.06 (0.83–1.37)	0.98 (0.77–1.24)
Transactional sex					
Young men	2.21 (0.84–5.87)	0.64 (0.16–2.52)	2.61 (0.87–7.80)	5.33*** (2.11–13.48)	0.07*** (0.02–0.27)
Young women	1.51 (0.67–3.41)	2.21 (0.70–7.02)	1.27 (0.41–3.87)	1.98 (0.82–4.81)	1.51 (0.58–3.94)

*aOR* age-adjusted odds ratio, *∞* Negligible data

*P < .05 **P < .01 ***P < .001

Similarly, social factors and HIV and other STI knowledge attenuated the relationship between media and contraceptive use; and after we accounted for HIV and other STI knowledge, those who did not read newspapers in Angola were 65% (aOR = 1.65 (1.26–2.15), p < 0.001), and in Nigeria were 85% (aOR = 1.89 (1.40–2.56), p < 0.001) more likely to use unreliable methods of contraception or not to use any form of contraception. Additionally, young men in Cameroon who did not own a phone were significantly less likely to engage in transactional sex (aOR = 0.53 (0.31–0.93), p = 0.02). With regards to traditional media use, young men in Mali who did not listen to radio were significantly less likely to have given cash or other gifts in exchange for sex (aOR = 0.41(0.18–0.94), p = 0.03). In contrast, their counterparts in South Africa were over five times (aOR = 5.33 (2.11–13.48), p < 0.001), and in Ethiopia were about four times more likely to engage in transactional sex (aOR = 3.60 (1.38–9.40), p = 0.009). On the other hand, young women in Nigeria who did not listen to the radio were 44% more likely (aOR = 1.44 (1.04–1.99), p = 0.02) to have engaged in transactional sex, and those who did not read newspapers and magazines were 77% more likely (aOR = 1.77 (1.67–2.69), p = 0.007) to have received cash, gift, or other materials in exchange for sex.

## Discussion

We examined the link between traditional and new media use and adolescent sexual risk behaviour, accounting for the socio-contextual factors that influence young people’s sexual and reproductive risk behaviour in Sub-Saharan Africa. We also considered the influence of HIV and other STIs knowledge in the association between media use and young people’s sexual risk behaviour. The findings of our study underscore the importance of simultaneously considering the social context in which young people are embedded and the synergistic influence of these social forces on sexual risk behaviour among young people. While accounting for the contextual factors and knowledge about HIV and other STIs knowledge attenuated the link between media use and some sexual risk behaviour in many Sub-Saharan African countries, both new media, and traditional media use continued to contribute to many sexual risk behaviours among young people. Despite the influence of these contextual factors on the link between media use and adolescent sexual risk behaviour, there remained remarkable between-country and regional differences in the link between media use and many sexual risk behaviours.

Our study reveals that having owned a mobile phone had the strongest independent influence on having multiple sexual partners in many countries, after accounting for these contextual factors and HIV and other STIs knowledge. This finding may be expected considering the proliferation of uncensored sexually explicit materials in Sub-Saharan Africa [16, 17, 19, 93]. In addition, those who did not have access to the internet in Mali had lower odds of having had two or more sexual partners. Hence, young people who do not have access to these new media sources may be protected from the uncontrolled availability of sexually explicit materials in the region. Moreover, lack of phone ownership and access to the internet limit networking and social connection among young people; lower connectivity between youths who lack these new media sources may reduce their number of sexual partners, which has been reported in previous studies [16, 31, 47]. Given that many intervention programs take advantage of the widespread use of new media by young people to provide accurate digital sexual and reproductive health information, young people who do not have phones or lack access to the internet may be deprived of accessing sexual health information. Additionally, the lack of phone ownership and access to the internet by youths relates to widespread poverty and skewed wealth distribution in Sub-Saharan Africa, which continue to prevent many young people from accessing digital health services [10, 49, 77].

Further, simultaneously accounting for contextual factors and HIV and other STIs knowledge lessened the odds of non-use or the use of unreliable contraception that are associated with lack of media use. However, the use of unreliable contraception or non-use of contraceptive methods remained prevalent in many countries because of a lack of access to both traditional and new media. Our findings about the high prevalence of unreliable contraception or non-use of contraceptive methods do not match available evidence about the impact of mass media communications on improving contraceptive use, and the progress that has been made in meeting the contraceptive needs of young people in Sub-Saharan Africa [28, 94, 95]. It is possible many young people may not be reached by programs that utilize digital and traditional media to disseminate sexual risk reduction interventions. Our findings corroborate other studies that document how poverty and sociocultural practices impede contraceptive uptake in parts of Nigeria and some Sub-Saharan African countries [96, 97]. Thus, there is a need for country-specific programs, increased health education efforts, reduction in the cost of modern contraceptive methods, and provision of youth-friendly health services to enhance contraceptive uptake among young people. Overall, the reduction in many sexual risk behaviours corroborates existing evidence that sexual and reproductive health interventions, community engagement, and collaborative efforts between national governments and international agencies in the region have contributed to reducing sexual risk behaviour among young people [94, 98].

Those who did not read newspapers in Cameroon were less likely to use unreliable contraception or not use any contraception. Similarly, boys in South Africa who did not read newspapers were less likely to engage in transactional sex. Cameroon and South Africa are highly globalized countries, and the use of the internet and phone ownership is remarkably high among young people. Since only a few youths read newspapers, the use of new media and other traditional media sources may exert a greater influence on sexual risk behaviour in those countries. On the other hand, boys in Mali and South Africa who listened to the radio had a higher likelihood of having exchanged cash or gifts for sex. These two countries are at various stages of globalization, with South Africa being a highly globalized country. On the other hand, a greater percentage of young people in Mali who participated in the survey listened to the radio, and it has been reported in previous studies that radio is commonly used among young people in Mali [99]. In addition, young people may tune on to erotic music on the radio, and they may view erotic scenes in newspapers and magazines which some studies link to a heightened risk of sexual risk behaviour [31, 100].

Similarly, young women in Nigeria who did not listen to the radio nor read newspapers and magazines were more likely to exchange sex for cash, gifts, or other material gratification. This finding could be because of the higher percentage of young people in Nigeria who did not have access to traditional media sources, and less than half of young people had access to the Internet. Poverty and the desire to own a phone may motivate girls and young women to seek cash, or other material gifts in exchange for sex [63, 101]. Our findings about the independent influence of media use on young people’s sexual risk behaviour match existing evidence that gaps remain in meeting global targets for adolescent sexual and reproductive risk behaviour in the region [102]. Thus, intervention programs should take advantage of the pervasive use of traditional and new media among young people in the region to provide accurate and comprehensive sexual and reproductive health education so that young people can make informed sexual health decisions. Our findings also emphasize the need to advocate for policies and legislation that will control young people’s access to inappropriate sexually explicit materials on both new and traditional media sources. Given that many countries in Sub-Saharan Africa are at various stages of globalization, country-specific programs, and intervention efforts will address the unique influences of both traditional and new media on youth sexual risk behaviour in the region. Finally, programs that aim to promote young people’s sexual and reproductive health in the region should consider the social inequities in phone ownership and access to the internet, as well as access to other media sources. This could guide the provision of targeted intervention to the most vulnerable groups. Programs and intervention efforts should leverage the proliferation of web-based applications and the use of both traditional and new media by young people to disseminate accurate and comprehensive sexual and reproductive risk prevention information to young people.

We acknowledge that our study used cross-sectional data and thus we do not claim a causal relationship between media use and youth sexual risk behaviour. We also recognize that the socio-contextual factors we included in our model may not be exhaustive, given the myriad of social factors, and socio-cultural practices in Sub-Saharan Africa that influence health behaviours among young people. For instance, peer and family relationships may influence media use and sexual risk behaviour. These areas are worth exploring in future studies. Our study involved youths, aged 15 to 24 years, not younger adolescents, because the Demographic and Health Surveys do not include those aged 10–14 years. Despite these limitations, our study provides population-based evidence about the role of several contextual factors in the link between traditional and new media use, as well as the gaps that remain in preventing sexual and reproductive risk behaviour among young people in Sub-Saharan Africa.

## Conclusion

Our findings reveal the importance of considering the embeddedness of young people within a wide array of intersecting socio-contextual factors that influence media use and engagement in sexual risk behaviour. We found that simultaneously accounting for socio-contextual factors and knowledge about HIV and other sexually transmitted infections attenuated the link between traditional and new media use and adolescent sexual risk behaviour in many Sub-Saharan African countries. However, media use (and lack of access to media) continues to be linked to sexual risk behaviour among young people in the region. Lack of access to traditional and new media sources links to unreliable contraception or non-use of any contraceptive methods. Further, young people, particularly boys who listened to the radio or read newspapers and magazines had higher odds of engaging in transactional sex in many countries. On the other hand, poverty, lack of access to sexual health information, and the desire to own mobile phones continue to expose young women to sexual risk behaviour. Young people who did not have mobile phones and lacked access to the internet were less likely to have multiple sexual partners in many countries of Sub-Saharan Africa. While progress has been made in reducing sexual risk behaviour among young people, gaps remain between countries and regions of Sub-Saharan Africa. These findings have implications for country-specific and tailored sexual and reproductive health intervention programs to meet the sexual and reproductive information needs of young people from diverse social backgrounds using both traditional and new media.

## Data Availability

Data that support the findings of the study are provided by the Demographic and Health Surveys (DHS) Program of the United States Agency for International Development and are available upon reasonable request and permission from the DHS Program. No datasets were generated or analysed during the current study.
